# Cardioprotection Achieved Through Overexpression of Relaxin Receptors∗

**DOI:** 10.1016/j.jacbts.2021.11.008

**Published:** 2022-01-24

**Authors:** Xiao-Jun Du

**Affiliations:** a Baker Heart and Diabetes Institute, Melbourne, Victoria, Australia; b Faculty of Physiology and Pathophysiology, College of Basic Medical Science, Xian Jiaotong University Health Science Center, Xian, China

**Keywords:** gene therapy, ischemia-reperfusion, relaxin, relaxin family peptide receptor, serelaxin

**Figure:**
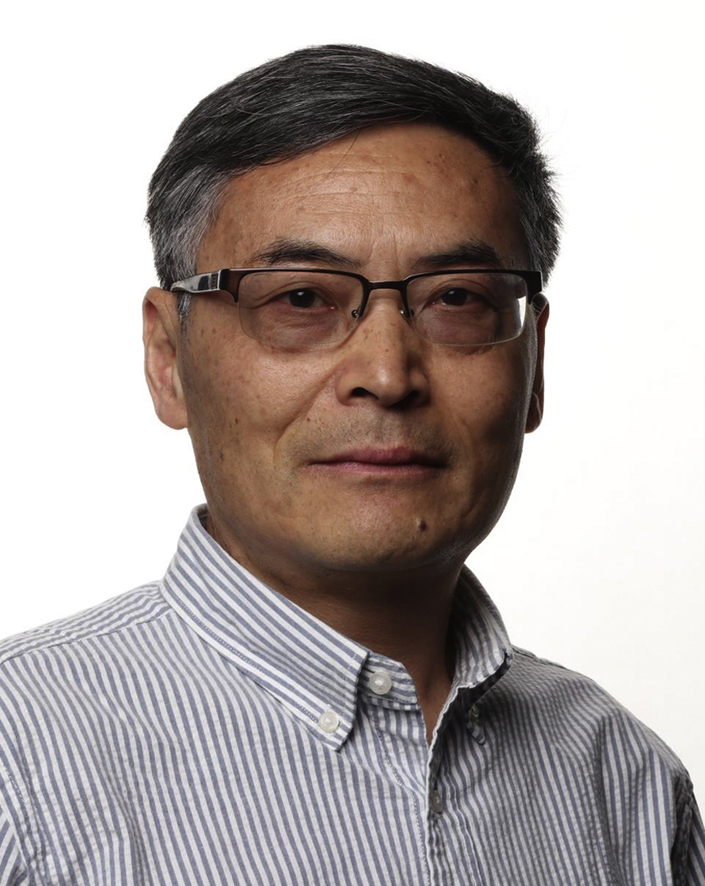

Relaxin is a naturally occurring peptide hormone that induces hemodynamic adjustment and matrix remodeling of reproductive organs during pregnancy. Whereas relaxin was discovered in 1926, its receptor, relaxin family peptide receptor (RXFP), was only identified in 2002.^1^ RXFP1 is widely expressed by cardiovascular cells and belongs to the family of G-protein-coupled receptors. Relaxin binds to RXFP1 with high affinity and activation of RXFP1 initiates a range of bioactivities through diverse signaling pathways.^1^ Previous studies have shown that relaxin possesses a broad array of cardiovascular actions in the settings of physiological and diseased conditions, particularly positive inotropy or chronotropy, vasodilatation, amelioration of ischemic injury, attenuation of inflammation, and antifibrosis actions.^1^ Preclinical studies conducted in a range of laboratory species have documented salutary effects of relaxin under conditions of ischemia-reperfusion (IR). Relaxin administration has been shown to limit infarct size, ameliorate inflammation and oxidative stress, improve angiogenesis, and reduce the extent of fibrosis.^1^^,^^2^

In this issue of *JACC: Basic to Translational Science*, Devarakonda et al^3^ studied virally mediated RXFP1 overexpression in the mouse heart by using adeno-associated virus-9 (AAV9)–RXFP1 at an optimized dose. They validated RXFP1 overexpression by assays of RXFP1 mRNA level and mitogen-activated protein kinases (p42/p44 MAPK, also called Erk1/2) as RXFP1 downstream target molecules, and by pressure-volume catheterization showing a sensitized cardiac inotropy in response to administration of serelaxin (recombinant human relaxin-2). The therapeutic potential of AAV9-RXFP1 gene transfection was then tested in mice injected with AAV9-RXFP1 4 weeks before IR (30-minute ischemia followed by reperfusion). Mice with RXFP1 overexpression exhibited smaller infarct size or scar size, measured at 24 hours or 4 weeks after IR, respectively, together with ameliorated left ventricular remodeling and better preservation of cardiac performance compared to controls. Moreover, using cardiomyocytes prepared from mice treated with AAV9-RXFP1 or control virus, and subjected to simulated IR, AAV9-RXFP1–treated cardiomyocytes further showed less cell death compared to control cells using trypan blue stain, a marker of necrotic cell death caused by loss of membrane integrity. Collectively, these findings suggest that forced overexpression of RXFP1 is sufficient to mediate cardioprotective signaling independent of exogenous relaxin. Although the exact signaling mechanism remains undefined, this study contributes to the field by showing that, in the setting of IR, cardioprotection against IR, as previously seen with the use of relaxin, could be achieved by RXFP1 overexpression.^2^

Devarakonda et al^3^ adopted AAV9 as the delivery vehicle to transfect cardiac cells with RXFP1 cDNA. Cardiomyocytes are likely the major cell type transfected by AAV9-RXFP1 because of a high affinity of AAV9 to striated muscle cells. RXFP1 overexpression in transfected heart tissues was validated by assay showing a marked increase in RXFP1 mRNA level. Whereas changes in myocardial RXFP1 at the protein level was undetermined, instead, the investigators compared difference in mice received the control virus or AAV9-RXFP1 using pressure-volume catheterization in functional response to relaxin in disease-free animals. Control mice responded only to subcutaneous injection of serelaxin at 1 mg/kg with an enhanced contractile force (eg, maximal systolic pressure, dP/dt_max_, measured at 15 minutes after relaxin administration), without change in stroke volume or heart rate. Overexpression of RXFP1 per se did not alter cardiac function at baseline, but augmented cardiac inotropy in response to serelaxin at 10 μg/kg to the level comparable to that in control mice in response to serelaxin at 1 mg/kg. Unexpectedly, attenuated ventricular relaxation (ie, reduction in both dP/dt_min_ and τ as the relaxation constant) was similarly observed in control mice (serelaxin 1 mg/kg) or in AAV9-RXFP1 treated mice (serelaxin 10 μg/kg). Previous studies in rats have shown increment of cardiac output and heart rate in vivo or in perfused heart.^1^^,^^2^ Clinically, in patients with acutely decompensated heart failure, administration of serelaxin (2 μg intravenous in bolus followed by 6-hour infusion at 4 μg/hour) reduced left ventricular preload (eg, pulmonary capillary wedge pressure), pulmonary arterial pressure, and systolic vascular resistance without change in cardiac output.^4^ Thus, in the setting of heart disease, the acute inotropic effect of relaxin-RXFP1, together with the reduction in cardiac preload and afterload, would be beneficial. To better determine the influence of overexpressed RXFP1 on cardiac inotropic and lusitropic responses to relaxin, without the influence of alterations in preload and afterload, cardiac function can be determined ex vivo in perfused hearts of animals with and without IR.

Numerous animal studies have well-documented the cardioprotection by relaxin therapy in the setting of IR or myocardial infarction. The findings made by Devarakonda et al^3^ raise an interesting question: How could overexpression of RXFP1 protect the heart against IR injury in the absence of exogenous relaxin? The intracellular signaling via RXFP1 is complex and involves diverse G-proteins (Gs, Go, and Gi) and multiple transduction pathways.^1^^,^^2^ One of the features of RXFP1 signaling is the assembly of signal protein complexes to form a RXFP1-signalosome, which sensitizes RXFP1 to even attomolar (10^-18^ M) concentration of relaxin, and activates downstream signaling involving Gs- and Gβγ-subunits with accumulation of cyclic adenosine monophosphate (cAMP).^5^ At higher concentrations of relaxin, RXFP1-signalsome is dissociated, allowing the activation of classical RXFP1 signaling pathways. The therapeutic implication of such biased signaling via RXFP1 by low concentrations of relaxin remains unclear.^5^ In healthy men and women, circulating relaxin levels are found to be within 2 and 100 ng/L, and such levels are expected to increase by a few fold under diseased settings, forming an endogenous source of RXFP1 ligand. Thus, it is likely that virally mediated overexpression of RXFP1 promotes formation of RXFP1-signalosome and “constitutive activity,” providing cardioprotection. Another feature of RXFP1 is its long-lasting signaling. In cells transfected with RXFP1 (assuming high levels of density), relaxin induces a prolonged signaling, such as accumulation of Gs-mediated cAMP lasting several hours. Indeed, Devarakonda et al^3^ found that RXFP1-transfected animals showed enhanced inotropic response upon administration of relaxin at a low dose (10 μg/kg subcutaneously in bolus) that failed to induce detectable functional change in control mice.^3^

Whereas the findings by Devarakonda et al^3^ bear therapeutic implication by enhancing RXFP1 expression, a few important issues remain undefined. First, previous studies showed satisfactory gene transfection of cardiomyocytes using AAV9, but it is unclear whether the insertion of a cardiomyocyte-specific promoter upstream of RXFP1 cDNA would ensure cardiomyocyte-targeted gene transfection, albeit the limited capacity of viral vector is a challenge. Nevertheless, similar to cardiomyocytes, other cardiovascular cell types (endothelial cells, smooth muscle cells, immune cells, or fibroblasts) are known to be equipped with RXFP1 that mediate beneficial signaling, and, when transfected with RXFP1, would be expected to contribute to the overall cardiac efficacy. Accordingly, it would be worthwhile to test the consequences of RXFP1 overexpression in various cell types in vitro and in vivo to observe cardiovascular protective actions. Second, Devarakonda et al^3^ did not validate an increased RXFP1 expression over control value at the protein level, which is apparently critical. Methods applicable to address this question include RXFP1 antibody-based assays (immunoblotting, immunohistochemistry), RXFP1 binding assay using ligands labeled with fluorescence or radioisotope, or bioassay of isolated cells to determine cAMP accumulation. Third, to gain insight on the significance of RXFP1 density per se on features of intracellular signaling, it would be interesting to conduct research on the relationship between RXFP1 density and intracellular signaling pattern(s) in response to relaxin in cardiovascular cells with overexpression of RXFP1. Further investigation on RXFP1 signaling is also warranted under ischemic or simulated ischemic conditions.

Among preclinical studies testing relaxin or serelaxin, therapeutic efficacy was achieved with a wide range of dosages tested (0.2 to 500 μg/kg/d). Using relaxin as a peptide drug has a few obvious drawbacks, such as that it has to be delivered via injection or intravenous infusion, its short-lasting effect, high cost of production, and its complex and nonlinear dose-effect relationship. AAV is one of the most actively investigated vehicles for gene therapy with merits of robust stability, efficiency, long-lived effect, and nontoxic nature. Currently, there have been approximately 200 clinical trials of gene therapy for heart disease using AAVs as the transgene delivery vehicle. The exciting findings by Devarakonda et al^3^ imply a new approach using RXFP1 overexpression for cardiotropic gene therapy. Despite disappointing outcomes of recent clinical trials on serelaxin therapy for acute decompensated heart failure, numerous preclinical studies have implicated beneficial effects of relaxin therapy on diverse cardiac diseases, most notably, inflammation, ischemic injury, and fibrosis.^2^ The relaxin-RXFP1–targeted therapy in these diseased conditions deserve further investigation for clinical translation.

## Funding Support and Author Disclosures

The author has reported that he has no relationships relevant to the contents of this paper to disclose.
